# A rodent obstacle course procedure controls delivery of enrichment and enhances complex cognitive functions

**DOI:** 10.1038/s41539-022-00134-x

**Published:** 2022-09-03

**Authors:** Sandra Gattas, Heather A. Collett, Andrew E. Huff, Samantha D. Creighton, Siobhon E. Weber, Shoshana S. Buckhalter, Silas A. Manning, Hardeep S. Ryait, Bruce L. McNaughton, Boyer D. Winters

**Affiliations:** 1 Department of Electrical Engineering and Computer Science, University of California, Irvine, CA USA; 2 Medical Scientist Training Program, University of California, Irvine, CA USA; 3 Department of Psychology and Collaborative Neuroscience Program, University of Guelph, Guelph, ON Canada; 4 Department of Biomedical Science, University of Guelph, Guelph, ON Canada; 5 Canadian Centre for Behavioural Neuroscience, University of Lethbridge, Lethbridge, AB Canada; 6 Department of Neurobiology and Behavior, University of California, Irvine, CA, USA

**Keywords:** Hippocampus, Cognitive ageing, Long-term memory

## Abstract

Enrichment in rodents affects brain structure, improves behavioral performance, and is neuroprotective. Similarly, in humans, according to the cognitive reserve concept, enriched experience is functionally protective against neuropathology. Despite this parallel, the ability to translate rodent studies to human clinical situations is limited. This limitation is likely due to the simple cognitive processes probed in rodent studies and the inability to control, with existing methods, the degree of rodent engagement with enrichment material. We overcome these two difficulties with behavioral tasks that probe, in a fine-grained manner, aspects of higher-order cognition associated with deterioration with aging and dementia, and a new enrichment protocol, the ‘Obstacle Course’ (OC), which enables controlled enrichment delivery, respectively. Together, these two advancements will enable better specification (and comparisons) of the nature of impairments in animal models of complex mental disorders and the potential for remediation from various types of intervention (e.g., enrichment, drugs). We found that two months of OC enrichment produced substantial and sustained enhancements in categorization memory, perceptual object invariance, and cross-modal sensory integration in mice. We also tested mice on behavioral tasks previously shown to benefit from traditional enrichment: spontaneous object recognition, object location memory, and pairwise visual discrimination. OC enrichment improved performance relative to standard housing on all six tasks and was in most cases superior to conventional home-cage enrichment and exercise track groups.

## Introduction

Decades of research findings support the conclusion that experience affects neural architecture and plasticity^1–5^. Indeed, enriched experiences have been shown to affect brain morphology and electrophysiology, promote positive effects on behavior, and can be protective against the effects of aging, Alzheimer’s disease (AD), other forms of dementia, and a myriad of additional disease states^6–14^. Although predominantly studied in animal models, similar findings have been reported in humans^15–17^, where factors including education level, vocabulary, hobbies, and multi-linguality serve as composite measures of “cognitive reserve”—an operational construct that has been posited to play a protective role against aging and dementia^18^.

Despite the overwhelming evidence regarding the beneficial effects of enrichment from rodent studies, and the cognitive reserve hypothesis pointing to promising protective effects in humans, there is limited translatability to human clinical situations. There are two aspects to the rodent environmental enrichment (EE) model that likely contribute to its limited clinical translatability.

First, most rodent studies have investigated enrichment effects on lower-order cognitive processes using multiple variants of the same simple behavioral tasks. These behavioral tasks primarily include finding a platform in a water maze (Morris Water Maze; MWM, probing spatial learning and memory), open field exploration (open field test; OFT, probing exploration and emotionality), elevated mazes (elevated plus maze; EPM, probing anxiety), and object recognition (OR, probing recognition memory)^19^. The output of this body of work is impressive and has established that EE improves spatial and non-spatial learning and memory, sensory discrimination, sociability, and anxiety, in wild-type rodents and/or models of aging and disease^6,19–26^. However, there is no fine-grained analysis of the effects of EE on complex aspects of cognition. This is needed for a better behavioral mapping between rodents and humans and will more clearly point to which aspects of complex mental disorders benefit from EE (or other forms of therapy). Here, we employ three new behavioral tasks probing different aspects of higher-order cognitive processes which are subject to deterioration due to aging and AD in humans^27–29^: (1) categorization and abstraction—hypothesized as the functional basis for the neocortical-hippocampal memory network in memory formation, consolidation, and retrieval^30,31^, (2) multisensory integration, which requires inter-regional communication, a prerequisite for engagement of the aforementioned network; and (3) higher-order perception.

Second, with existing methods of enrichment, it is not possible to control for the degree of animal engagement with enrichment stimuli or identify which aspects of enrichment contribute to the measured outcomes. It is important to have a method which controls for animal engagement with objects to study how factors such as age, disease, or brain manipulations interact with EE effects, because such variables can influence animals’ motivation level and, therefore, receipt of enrichment. Moreover, despite recent improvements in standardization of enrichment methods^32,33^, as opposed to traditional enrichment (placing animals in larger cages, group-housed, with extra bedding, toys, and running wheels), there is not an existing method that enables parsing out social stimulation, cognitive stimulation, and physical exercise. Here, we present an enrichment paradigm, the Obstacle Course, which addresses these two foregoing limitations to existing enrichment methods.

**Fig. 1:**
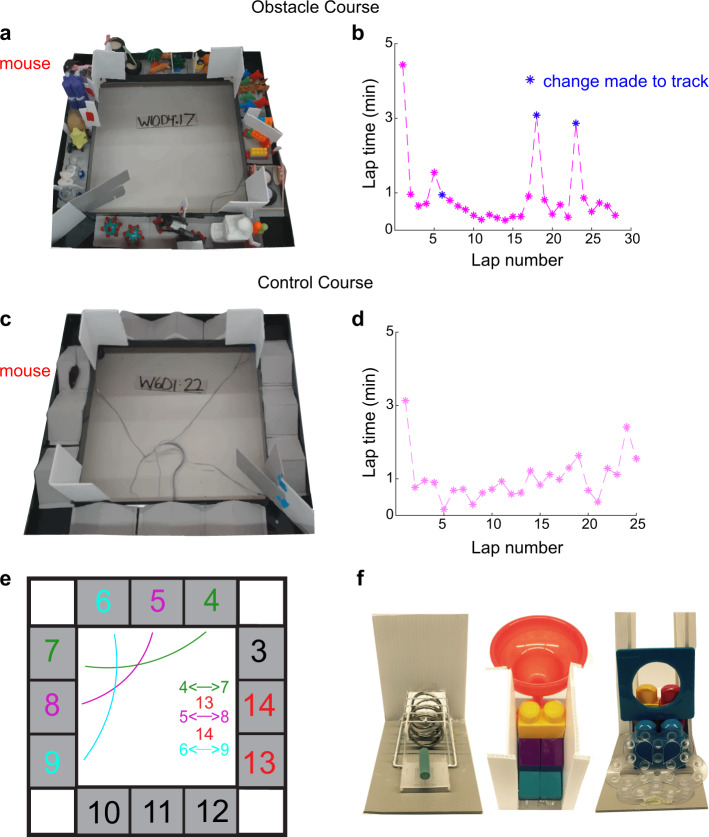
The obstacle course paradigm. **a** Example OC track filled with 12 inserts. **b** Example lap time data for a 30-min duration on the OC. Note that animals tended to take longer to complete a given lap when a change to the track was performed immediately prior to the lap (blue asterisk). See Supplementary Video 2 for a demonstration of the animal change in speed pre and post-change to the track. **c** An example CC track filled with 12 hurdles. **d** Example lap time data for a 30-min duration on the CC. No changes were made to the CC throughout a given session. **e** Schematic of changes made in a 1-h OC session. A total of 5 changes were made, which included three rearrangements (e.g., swapping obstacle 4 with obstacle 7, indicated by arrows) interleaved with two replacements of old inserts with new ones (indicated in red). See Supplementary Fig. 1 for a detailed OC and CC protocol. **f** Three example obstacles used in the OC (12 for the entire course).

We advance the EE model’s utility in two ways: (1) showing that enrichment also enhances specific aspects of higher-order cognitive functions in mice by using a series of complex behavioral tasks, and (2) delivering enrichment through a new paradigm, the Obstacle Course (OC), which enables systematic control over animal engagement with enrichment stimuli, is delivered outside of animal housing and has an exercise-matched Control Course (CC) (Fig. 1). We tested enrichment effects on categorization, perceptual invariance, and multisensory integration using new paradigms for object category, view-invariance, and cross-modal object recognition, respectively (Table 1). We found that OC training elicited improved and long-lasting performance on the three aforementioned tasks, especially at longer delays between encoding and retrieval. These effects were not observed with exercise alone, tested using the CC. Lastly, because these enrichment effects were observed with our OC enrichment paradigm, we also validated the OC by replicating findings obtained from three previously utilized tasks: object recognition, object location memory, and pairwise visual discrimination. For all tasks, enrichment was delivered both through the OC and through traditional enriched housing (EH). The OC group was comparable to the EH group on tasks with shorter learning-to-testing delays and outperformed the EH group on most tasks with longer delays.

**Table 1:** Experimental timeline.

Event	Date	Age of mice	Duration since the discontinuation of enrichment
First day of enrichment	10/08/18	1.5 m	—
Last day of enrichment	12/15/18	4 m	—
First day of SOR testing during EE	12/3/18	4 m	0
Last day of SOR testing during EE	12/15/18	4 m	0
SOR rep post-EE	01/12/19	5 m	28 d [4 wks]
SOR rep post-EE	01/15/19	5 m	31 d [4.4 wks]
First day of OCR testing	02/01/19	6 m	48 d [6.8 wks]
Last day of OCR testing	03/01/19	6 m	75 d [10.7 d]
First day of VIOR testing	03/06/19	7 m	81 d [11.6 wks]
Last day of VIOR testing	03/20/19	7 m	95 d [13.6 wks]
First day of CMOR testing	04/10/19	8 m	116 d [16.6 wks]
Last day of CMOR testing	04/23/19	8 m	129 d [18.4 wks]
First day of OL testing	05/06/19	9 m	142 d [20.3 wks]
Last day of OL testing	06/16/19	10 m	183 d [26.1 wks]
First day of PD testing	06/24/19	10 m	191 d [27.3 wks]
Last day of PD testing	8/11-10/19	14–16 m	231 d [33 wks]

Table outlining enrichment and behavioral testing schedule, including experiment date, age of mice during each experiment, and duration between enrichment discontinuation and behavioral testing.

*rep* repeated, *m* months, *wks* weeks, *d* days.

## Results

For all object-based tasks, object exploratory behavior is reported, and significant results are summarized in the supplementary materials (Supplementary Tables 1, 2).

### Spontaneous object recognition (SOR): enhancement of long-term object memory with OC training

**Fig. 2:**
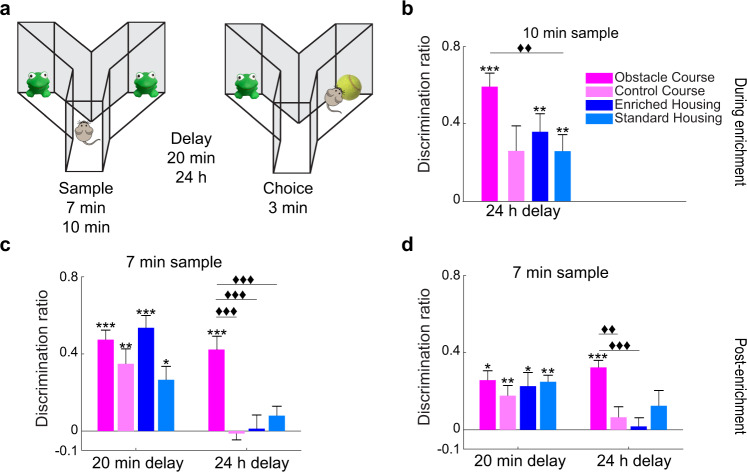
SOR—Enhancement of long-term object memory with OC training exceeds EH effects. **a** Schematic representation of the spontaneous object recognition (SOR) experiments. **b** Task performance with a 10-min sample and a 24-h delay during the 9th week of enrichment. The OC group demonstrated a significantly higher discrimination ratio (DR) compared to the SH group. OC, EH, and SH groups showed DRs above chance. **c** SOR task performance with a suboptimal sample time at two delay durations during the 10th week of enrichment. With a 20-min delay, all groups significantly discriminated above chance. With an increased delay to 24 h, the OC group DR was significantly higher compared to all other groups. (Delay x group interaction *p* < 0.001, delay main effect *p* < 0.001, and group main effect *p* = 0.003). Only the OC group showed a DR above chance. **d** Replication of c, but completed 1-month post-discontinuation of enrichment. With a 20-min delay, all groups significantly discriminated above chance, demonstrating intact object memory. With an increased delay to 24 h, the OC group demonstrated enhanced object memory, performing significantly better than the EH and CC groups (delay x group interaction *p* = 0.057, delay main effect *p* = 0.01, and group main effect *p* = 0.023). The OC group was the only group to discriminate the novel object significantly above chance. *p < 0.05, **p < 0.01, ***p < 0.001 indicate significant two-tailed paired-sample t-tests (choice DR vs. sample DR). ♦♦*p* < 0.01, ♦♦♦*p* < 0.001 indicate a significant difference between groups (two-tailed independent samples *t*-test, Bonferroni correction *p* = 0.0083). Error bars represent the standard error of the mean (s.e.m.) across animals.

We first tested whether the OC experience can induce the previously reported EE enhancements of object recognition memory^34,35^. Four groups of mice, Obstacle Course (OC), Control Course (CC), Enriched housing (EH), and Standard housing (SH), were first tested on the SOR task with a 10-min sample phase and a 24-h delay in the 9th week of enrichment (Fig. 2a, b). In this first experiment, an analysis of variance (ANOVA) showed that the effect of group was not significant, *F*(3, 36) = 2.613, *p* = 0.066, partial *η*^2^ = 0.179. However, planned comparisons using independent samples *t*-tests indicated that the OC group showed an enhancement in task performance compared to the SH group, *t*(18) = −3.027, *p* = 0.007, after Bonferroni correction for multiple comparisons (*p* = 0.0083). Two-tailed paired-samples *t*-tests (sample DR vs. choice DR) showed that mice in the OC, *t*(9) = −8.109, *p* < 0.001, EH, *t*(9) = −3.624, *p* = 0.006, and SH groups, *t*(9) = −3.846, *p* = 0.004, all demonstrated evidence of intact memory.

In a subsequent experiment, we introduced both short and long-term retention delays and increased task difficulty by shortening the sample phase to 7 min (Fig. 2a, c). A repeated measures ANOVA revealed a significant interaction between length of delay and group, *F*(3, 36) = 16.818, *p* < 0.001, partial *η*^2^ = 0.584. Significant main effects were also found for delay, *F*(1, 36) = 124.724, *p* < 0.001, partial *η*^2^ = 0.776, and group, *F*(1, 36) = 5.720, *p* = 0.003, partial *η*^2^ = 0.323. With the 20-min delay, differences between groups were not significant and a two-tailed paired-samples *t*-test showed that the OC, *t*(9) = −7.364, *p* < 0.001, CC, *t*(9) = −4.784 *p* = 0.001, EH, *t*(9) = −11.927, *p* < 0.001, and SH groups, *t*(9) = −3.114, *p* = 0.012, all demonstrated intact object memory. However, between-group differences were observed with the 24-h delay. Further analysis with post hoc independent samples *t*-tests revealed significant differences in performance between the OC group and the EH group, *t*(18) = −6.287, *p* < 0.001, CC group, *t*(18) = −5.573, *p* < 0.001 and SH group, *t*(18) = −5.836, *p* < 0.001, when the delay was 24 h. Two-tailed paired-samples *t*-tests showed that only the OC group discriminated the novel object *t*(9) = −8.075, *p* < 0.001.

One month following the end of the enrichment period, the enhancement observed on the SOR task with a 7-min sample and 24-h delay persisted for the OC group (Fig. 2a, d). Observed were significant main effects of group, *F*(3, 36) = 3.575, *p* = 0.023, partial *η*^2^ = 0.230, and delay, *F*(1, 36) = 7.421, *p* = 0.010, partial *η*^2^ = 0.171, while the interaction between group and delay was not significant, *F*(3, 36) = 2.745, *p* = 0.057, partial *η*^2^ = 0.186. Further analysis with planned independent samples *t*-tests revealed a significant difference in performance on the task between the OC group and the EH group, *t*(18) = −5.292, *p* < 0.001 and the CC group, *t*(18) = 3.917, *p* = 0.001 when the delay was 24 h. At this delay, only the OC group performed significantly above chance, *t*(9) = −5.962, *p* < 0.001. When the delay was reduced to 20 min, each group’s performance, OC, *t*(9) = −2.750, *p* = 0.022, CC, *t*(9) = −4.397, *p* = 0.002, EH, *t*(9) = −2.773, *p* = 0.022, and SH, *t*(9) = −4.054, *p* = 0.003, was above chance on the SOR task.

### Object location memory (OLM): enhanced object spatial memory with OC training

**Fig. 3:**
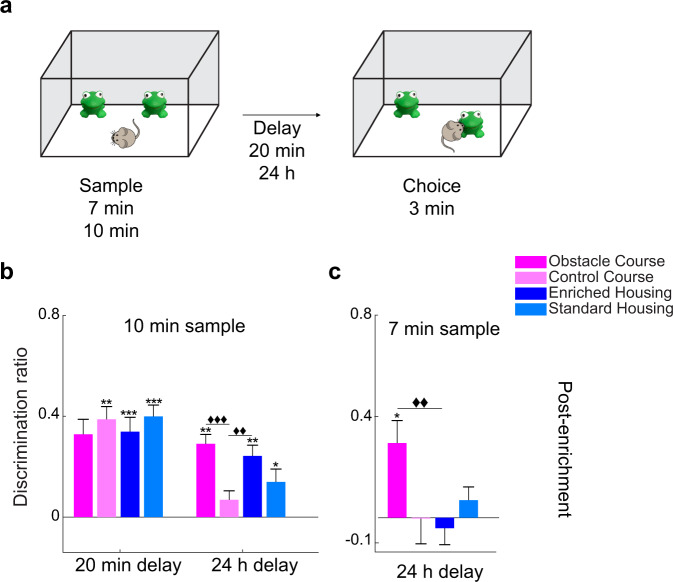
OLM: enhanced memory for the location with OC training exceeds EH effects. **a** Schematic of the object location memory (OLM) experiments. **b** OLM task performance. With a 20-min delay, the CC, EH, and SH groups demonstrated intact spatial memory. With a 24-h delay, the OC and EH groups demonstrated enhanced object location memory, performing significantly better than the CC group (delay x group interaction *p* = 0.011, delay main effect *p* < 0.001, and group main effect *p* = 0.421). The OC, EH, and SH groups discriminated the displaced object above chance. **c** Performance with a 7-min sample and 24 h delay. The OC group showed enhanced memory relative to the EH group (Group main effect *p* = 0.022). Only the OC group discriminated significantly above chance. The experiment was implemented 5–6.5 months post enrichment. *p < 0.05, **p < 0.01, ***p < 0.001 indicate significant two-tailed paired-sample *t*-tests (choice DR vs. sample DR). ♦♦*p* < 0.01, ♦♦♦*p* < 0.001 indicate a significant difference between groups (two-tailed independent samples *t*-test, Bonferroni correction *p* = 0.0083). Error bars represent s.e.m. across animals.

For the second validation experiment, we investigated whether OC training can elicit enhancements in spatial memory as previously demonstrated with EE^36^, and more specifically, object location enhancements^34^. To do so, all groups performed the OLM task (Fig. 3a). Enhanced task performance after OC training was observed when the delay between sample and choice was increased from 20 min to 24 h (Fig. 3b). With the same sample time of 10 min, a repeated measures ANOVA revealed a significant interaction between the length of delay and group, *F*(3, 36) = 4.294, *p* = 0.011, partial *η*^2^ = 0.264, and a significant main effect of delay, *F*(1, 36) = 30.763, *p* < 0.001, partial *η*^2^ = 0.461; however, no main effect of group, *F*(3, 36) = 0.963, *p* = 0.421, partial *η*^2^ = 0.074 was found (Fig. 3b).

With a 20-min delay, differences between groups were not significant (Fig. 3b). Two-tailed paired-samples *t*-tests (sample DR vs. choice DR) showed that the CC, *t*(9) = −5.279, *p* = 0.001, EH, *t*(9) = −5.394, *p* < 0.001, and SH groups, *t*(9) = −6.056, *p* < 0.001, all discriminated the displaced objects, while discrimination by the OC, *t*(9) = −2.224, *p* = 0.053, was not significant. Subsequent planned analysis for the 24-h delay using independent samples *t*-tests revealed significant differences in performance on the task between the OC and CC groups, *t*(18) = 4.340, *p* < 0.001, as well as the EH and CC groups, *t*(18) = 3.136, *p* = 0.006. Moreover, with this longer delay of 24-h, the enriched groups, OC, *t*(9) = 5.092, *p* = 0.001 and EH, *t*(9) = 4.464, *p* = 0.002, and the SH group, t(9) = 2.746, *p* = 0.023, discriminated the displaced object.

Lastly, to probe the differences in spatial memory between the two enrichment groups further, we tested all groups with a shortened sample time of 7 min and a delay duration of 24 h. In this experiment, an ANOVA showed a significant effect of group, *F*(3, 36) = 3.623, *p* = 0.022, partial *η*^2^ = 0.232, and independent samples *t*-tests revealed that the OC group showed an enhancement on task performance compared to the EH group, *t*(18) = −3.041, *p* = 0.007 (Fig. 3c). At this suboptimal sample and increased delay, only mice in the OC group, *t*(9) = 2.585, *p* = 0.029 discriminated the displaced object.

### Pairwise visual discrimination (PD): enhanced cognitive flexibility with OC and CC training

**Fig. 4:**
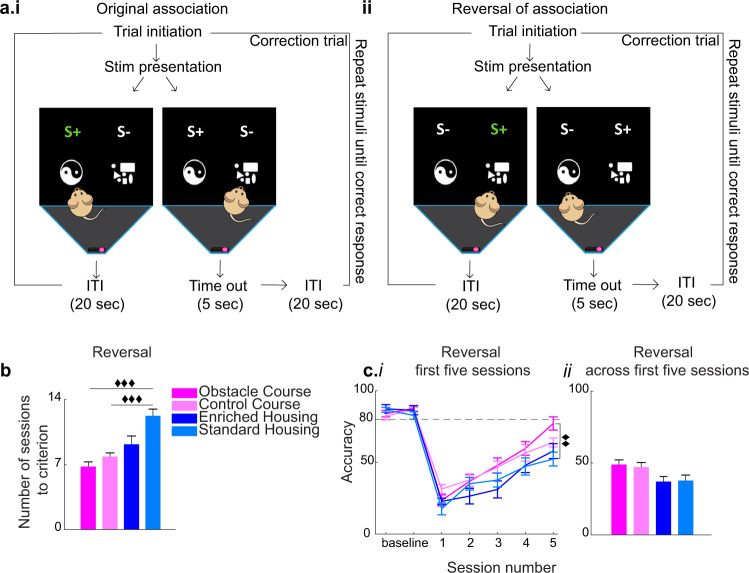
PD task—enhanced cognitive flexibility with OC and CC training exceeds EH effects. **a** Schematic representation of the pairwise visual discrimination (PD) experiment. **ai-ii** Stimulus-reward association learning. **ai** Original association: animal receives reward upon contact with the S+ stimulus. **aii** Reversal of association: previously learned S− stimuli become S+ stimuli and are associated with reward. **b** Mean session number to reach criterion (performance of 80% accuracy on two successive sessions) in the reversal phase per group (group effect *p* < 0.001), with the OC (*p* < 0.001) and CC (*p* < 0.001) groups requiring significantly fewer sessions compared to the SH group. **ci** Group percent accuracy on the first five reversal sessions, with a main effect of session (*p* = 0.003) and an effect of group on the fifth session (*p* < 0.001), with the OC group performing significantly higher than the SH group on session five. **cii** Mean accuracy across the first five reversal sessions for each group, with a group main effect (*p* = 0.034). Post hoc independent samples *t*-test did not reveal group pairwise differences. ♦♦*p* < 0.01, ♦♦♦*p* < 0.001 indicate a significant difference between groups (two-tailed independent samples *t*-test, Bonferroni correction *p* = 0.0083). Error bars represent s.e.m. across animals.

For the last replication experiment, we validated whether the OC experience can induce reported EE effects outside the domain of object-based tasks. It was previously shown that the PD task is effective in assessing cognitive function between groups (i.e., sex, disease models^37^) and that EE enhances reversal learning in this task^38^. Differences between groups were not significant in the number of pretraining days *F*(3,36) = 1.928, *p* = 0.142, partial *η*^2^ = 0.138 (habituation stages 1–7), nor in the acquisition phase (in regard to overall accuracy defined as a percent of correct trial responses, number of sessions to criterion and session accuracy) (Supplementary Fig. 2). Additionally, no group main effects were observed for acquisition and reversal trial touch and reward collection latencies (Supplementary Fig. 3). With regard to the reversal phase (Fig. 4a.ii), there was a group main effect in the number of sessions to reach criterion (achieving 80% accuracy or higher on two successive sessions), *F*(3,36) = 12.819, *p* < 0.001, partial *η*^2^ = 0.517 (Fig. 4b), and independent samples *t*-tests revealed significant differences between the OC and SH groups, *t*(18) = −6.104, *p* < 0.001 as well as the CC and SH groups *t*(18) = −5.273, *p* < 0.001, whereby the OC and CC groups required significantly fewer sessions. Additionally, there was a main effect of session when considering mean group accuracy in each of the first five sessions (where the first mouse reached criterion), *F*(3,36) = 61.723, *p* < 0.001, partial *η*^2^ = 0.632 (Fig. 4c.i), a main effect of group *F*(3,36) = 3.209, *p* = 0.034, partial *η*^2^ = 0.211, but the interaction between group and session was not significant *F*(3,36) = 1.045, *p* = 0.411, partial *η*^2^ = 0.080. Post hoc analysis of session performance revealed an effect of group during session 5, *F*(3,36) = 5.606, *p* = 0.003, partial *η*^2^ = 0.318. Independent sample *t*-tests revealed that the OC group performed with significantly higher accuracy compared to the SH group on session five, *t*(18) = 3.719, *p* = 0.002 (Fig. 4.c.i). Lastly, there was an effect of group when considering mean accuracy across the first five sessions, *F*(3,36) = 3.222, *p* = 0.034, partial *η*^2^ = 0.212 (Fig. 4c.ii).

### Object category recognition (OCR): enhancement of object category recognition with OC training

**Fig. 5:**
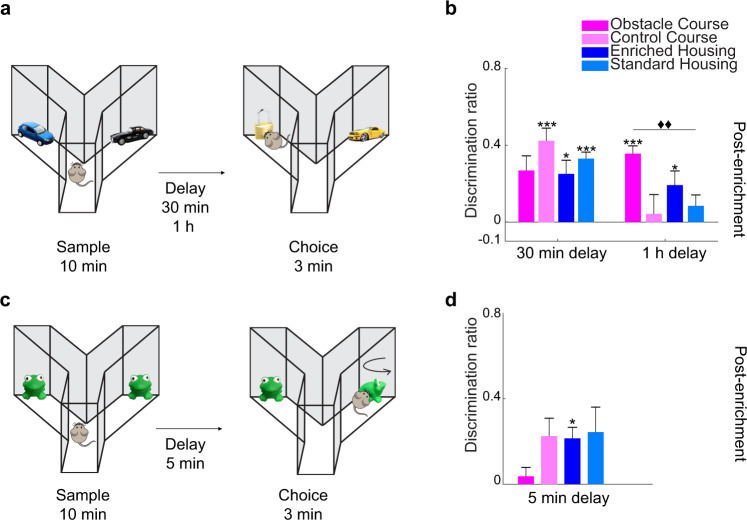
OCR and VIOR—Enhancement of both object category recognition (OCR) and object recognition despite rotational changes to objects (VIOR) with OC training. **a** Schematic representation of the object category recognition (OCR) experiment. **b** OCR task performance with two delay durations. With a 30-min delay, the CC, EH, and SH groups significantly discriminated above chance, demonstrating intact object category memory. With an increased delay to 1 h, the OC group demonstrated enhanced object category memory, performing significantly higher compared to the SH group (Delay x group interaction *p* = 0.001, delay main effect *p* = 0.001, and group main effect *p* = 0.532). The OC and EH groups significantly discriminated the novel category above chance. OCR experiments were implemented 1.5–2.5 months post-discontinuation of enrichment. **c** Schematic representation of the view-invariant object recognition (VIOR) experiment. **d** VIOR task performance. The OC group treated the rotated object as familiar, equally exploring rotated, and unrotated objects in the choice phase. The EH group significantly discriminated the rotated object. No significant group effect was observed (*p* = 0.240). VIOR experiments were implemented 3 months post-discontinuation of enrichment. *p < 0.05, **p < 0.01, ***p < 0.001 indicate significant two-tailed paired-sample *t*-tests (choice DR vs. sample DR). ♦♦*p* < 0.01, ♦♦♦*p* < 0.001 indicate a significant difference between groups (two-tailed independent samples *t*-test, Bonferroni correction *p* = 0.0083). Error bars represent s.e.m. across animals.

Next, we examined OC enrichment effects on arguably more complex cognitive domains than those previously explored. First, we tested whether the OC enrichment enhances object category recognition memory (Fig. 5a)^39^. All groups were tested on two retention delays (30 min, 1 h), both with a 10-min sample. A repeated measures ANOVA revealed a significant interaction between length of delay and group, *F*(3, 36) = 7.023, *p* = 0.001, partial *η*^2^ = 0.369, and a significant main effect for delay, *F*(1, 36) = 14.594, *p* = 0.001, partial *η*^2^ = 0.288, but not for group, *F*(1, 36) = 0.745, *p* = 0.532, partial *η*^2^ = 0.058 (Fig. 5b). With the 30-min retention delay, two-tailed paired-samples *t*-tests (sample DR vs. choice DR) showed that mice in the CC, *t*(9) = −6.135, *p* < 0.001 EH, *t*(9) = −3.129, *p* = 0.012, and SH groups, *t*(9) = −6.074, *p* < 0.001 all demonstrated intact object category memory, while the OC, *t*(9) = −1.876, *p* = 0.093, performance was not significant. However, between-group differences were observed when the retention delay was increased to 1 h (Fig. 5b). Further analysis with planned comparisons using independent samples *t*-tests revealed a significant difference in group performance on the task between the OC and the SH group, *t*(18) = 3.884, *p* = 0.001, when the delay was 1 h. Two-tailed paired-samples *t*-tests showed that the OC *t*(9) = −5.895, *p* < *0.001* and the EH *t*(9) = −2.937, *p* = 0.017, groups discriminated the novel category.

### View-invariant object recognition (VIOR): enhancement of object recognition despite rotational changes in object orientation

We then investigated the effects of OC enrichment on higher-order perception using VIOR testing (Fig. 5c). An ANOVA showed that the effect of group was not significant, *F*(3, 36) = 1.468, *p* = 0.240, partial *η*^2^ = 0.109. The OC group, to the most extent (numerically), treated both rotated and non-rotated objects as familiar, exploring them equally (Fig. 5d). Conversely, a paired-samples *t*-test showed that the EH group displayed a preference for the rotated object, suggesting that this group tended to perceive the rotated object as novel *t*(9) = −3.046, *p* = 0.014.

### Cross-modal object recognition (CMOR): enhanced cross-modal object memory with OC training

**Fig. 6:**
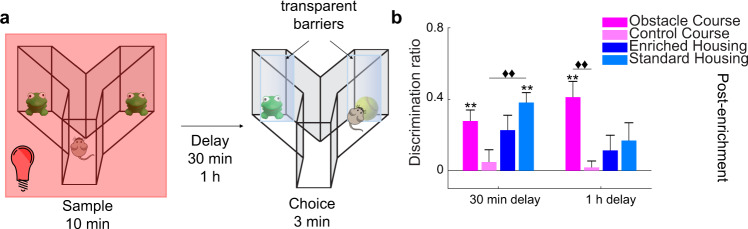
CMOR: enhanced cross-modal object memory with OC training. **a** Schematic of the cross-modal object recognition (CMOR) experiment. **b** CMOR task performance. At a 30-min delay, the SH group discriminated significantly more than the CC group. The OC and SH groups discriminated the novel object above chance. With a 1-h delay, the OC group demonstrated enhanced performance relative to the CC group (Delay x group interaction *p* = 0.056, delay main effect *p* = 0.216, and group main effect *p* = 0.005). Only the OC group could discriminate the novel object. The experiment was implemented 4–4.5 months post enrichment. *p < 0.05, **p < 0.01, ***p < 0.001 indicate significant two-tailed paired-sample *t*-tests (choice DR vs. sample DR). ♦♦*p* < 0.01, ♦♦♦*p* < 0.001 indicate a significant difference between groups (two-tailed independent samples *t*-test, Bonferroni correction *p* = 0.0083). Error bars represent s.e.m. across animals.

Lastly, we identified the degree to which OC enrichment facilitates the integration of sensory information from different modalities by testing all groups on a tactile-to-visual CMOR task (Fig. 6a). Enhanced task performance after OC training was observed when the delay between sample and choice was increased to 1 h (Fig. 6b). A repeated measures ANOVA did not show a significant main effect of delay, *F*(1, 36) = 1.584, *p* = 0.216, partial *η*^2^ = 0.042, and the interaction between length of delay and group was not significant *F*(3, 36) = 2.762, *p* = 0.056, partial *η*^2^ = 0.187. However, there was a significant main effect of group *F*(3, 36) = 4.981, *p* = 0.005, partial *η*^2^ = 0.293.

At a 30-min delay, planned independent samples t-tests revealed a significant difference in group performance on the task between the CC and the SH group, *t*(18) = −3.758, *p* = 0.001 (Fig. 6b). At the same delay, two-tailed paired-samples *t*-tests showed that both the OC, *t*(9) = −3.720, *p* = 0.005, and the SH, *t*(9) = −4.468, *p* = 0.002 groups discriminated between the novel and familiar objects. Subsequent planned analysis for the 1-h delay using independent samples t-tests revealed a significant difference in group performance on the task between the OC and the CC group, *t*(18) = 4.119, *p* = 0.001. Moreover, at this 1-h delay, only the OC group discriminated between the novel and familiar objects *t*(9) = −4.357, *p* = 0.002.

## Discussion

In the present study, we sought to advance the rodent enrichment model by performing a fine-grained analysis on the effects of EE on different aspects of higher-order cognition using the OC as a new protocol enabling controlled delivery of enrichment. We first showed that the OC is at least comparable or superior to traditional home-cage enrichment in terms of the benefits conferred on performance in established learning and memory tasks. Then we presented an expanded account of enrichment-mediated enhancements of lesser studied cognitive functions: object categorization/generalization, object perceptual processing, and information transfer across sensory networks. The extension of enrichment effects to these domains should help to improve behavioral mapping between rodents and humans in that they are more complex functions associated with deterioration from aging and dementia^27–29^. This kind of fine-grained, systematic analysis using new behavioral tasks will be critical in revealing which aspects of complex mental disorders could benefit from enrichment or other forms of therapy. The present study takes important first steps in revealing the potential for beneficial effects of enrichment interventions across a spectrum of complex cognitive abilities. Lastly, delivery of enrichment in a controlled manner, accomplished with the OC protocol, can provide greater insight into which aspects of enrichment and how the degree of engagement with stimuli influences certain processes.

Our results were observed with a new enrichment paradigm, the Obstacle Course. Conversely, Enriched Housing did not elicit improvements in view-invariant object processing nor cross-modal sensory integration and yielded a less prominent effect on category recognition. The OC group also outperformed current standards of enrichment on previously reported measures, demonstrating superior long-term memory for object identity and spatial location (especially with short, ‘suboptimal’ study durations) and enhanced cognitive flexibility (pairwise discrimination reversal). Most of these effects cannot be explained by physical exercise alone, as tested through an exercise-matched Control Course. Lastly, OC-induced enhancements were sustained for over 8 months after discontinuation of enrichment, an important demonstration of the potential longevity of effects produced by this protocol.

Across tasks, the main enhancement effects with OC training that significantly differed from EH were generally observed with shorter sample times and longer delays (SOR, OCR, CMOR, and OL). This suggests that, with OC training, less time is necessary to encode and/or consolidate information about the external environment into long-term memory. In line with this, is the observed result of superior performance by the OC group compared to all other groups on OCR. We have previously reported that mice can only perform OCR with retention delays longer than 30 min when an exemplar preexposure period is implemented, which possibly enables an improved representation of the to-be-remembered object categories^39^. However, in the current study, the OC group was able to perform the task successfully without an exemplar preexposure period. Thus, the benefit of enrichment in general, and our OC paradigm in particular, could be explained at least partly by improved schema formation.

Schema formation is a process reliant on semanticization and abstraction of experiences and generation of internal representations which are categorically organized. The facilitation of these processes could enable items to be more rapidly integrated into long-term memory if they are to some degree consistent with existing memories or schemas^30,31,40^. The OC experience requires rich interactions with a wide variety of object-based obstacles, which may facilitate the establishment of object schemas. Thus, a reasonable hypothesis is that OC animals have more highly diversified internal representations of the world that would support such an effect. This possibility, as well as alternatives involving enhanced learning and memory via boosting of neuroplasticity mechanisms, warrant further testing in subsequent studies to unravel the means by which OC training achieves superior effects.

OC training also improved perception and multisensory integration. With OC training, mice recognized object identity despite rotation (VIOR) to a greater degree than observed with EH. This finding also supports the notion that OC mice generated a stronger internal representation and/or better-retained object representations, recognizing a given object from a different angle. Another possibility is that more sophisticated perceptual processes were at play and not merely superior long-term memory^41,42^. Additionally, with OC enrichment, mice were better able to perform an explicitly cross-modal cognitive task, visually recognizing objects that they had previously only explored tactilely. The mechanisms by which these enhancements occurred cannot be delineated from the present study. However, these results demonstrate that OC training enhances neural processes that can be impaired in human cognitive disorders. Tasks such as OCR, VIOR, and CMOR probe how animals integrate sensory information about object features to build, maintain and utilize object representations. Supporting the translational value of using these tasks, deficits in feature binding and multisensory integration can be observed in human disorders such as AD^43^, schizophrenia^44^, and autism spectrum disorder^45,46^, and have also been observed in animal models of these disorders^47–49^. In particular, the binding of object features has been shown to be impaired in the early stages of AD^50^, but preserved with healthy aging in humans^51^ and rodents^52^. Accordingly, neuropathological, structural and electrophysiological studies indicate impaired inter-regional communication in AD; for example, neuropathology is concentrated in areas of cortico-cortical and neocortical-hippocampal exchange as well as heteromodal association cortices^28^. Altogether, OC enrichment in the current study appeared to enhance neural processes in rodents that are at-risk with aging or AD, as well as other human disorders of cognition characterized by dysfunction in distributed brain networks.

It should be noted that exploration time analyses support the notion that OC enhancements are not explained by influences on animals’ longer exploration durations with novel objects. While significant differences in exploration time were detected for each of the four groups, the direction of such differences did not display a straightforward pattern (Supplementary Table 1) and were not linearly associated with discrimination ability (Supplementary Table 2). The reported OC-mediated enhancements also cannot be ascribed to reward type (milkshake for OC/CC vs. peanut butter for EH) because in half the cases where significant OC effects are reported, OC performance was significantly better than CC performance, and in the other half of the cases, enhancements were not seen in the CC group, despite both groups receiving the same reward. Indeed, the provision of strawberry milkshake versus peanut butter introduces a difference in diet between groups. We therefore cannot fully eliminate the possibility that the provision of peanut butter masked home-cage enrichment-mediated enhancements. Nevertheless, this confound in the diet does not diminish our report of enrichment-mediated higher-order cognitive enhancements and the methodological advance of enrichment delivery using the OC.

A few additional clarifications regarding the present study are worth noting. First, the Standard Housing group performed significantly better compared to the Control Course group on the CMOR task. This could possibly be explained by the potential adverse effects of removal of physical exercise from the CC group, which was not experienced by the SH group. Rather, the SH group only experienced positive changes as it was moved into slightly larger cages post completion of the enrichment protocols. Secondly, while group means trended towards improved performance by the OC group compared to all others on several aspects of PD task acquisition, we did not find robust differences in performance between OC and EH. This is consistent with a prior report of EE primarily enhancing reversal learning compared to aspects of the acquisition phase in this task^53^. This could be related to the longitudinal nature of testing required in the present study; all groups may have benefitted as testing continued following the explicit enrichment phase^54^. Alternatively, it is possible that OC training produces differential effects on different brain circuitry, the requirements of which vary across behavioral tests—this is an intriguing possibility that requires further investigation. Indeed, additional work is needed to clarify the specific neurobiological effects of OC and CC training and how these might differ from traditional enriched housing conditions. Lastly, due to the labor-intensive nature of running each animal daily for an hour, we were limited in the number of mice per group (*n* = 10 mice, *n* = 2 groups, 20-h per day for 20 mice). Given this practical limitation and the abundance of male-only studies in the EE literature, we opted to test male mice only in this initial study. While we predict that female mice would undergo similar OC-mediated cognitive enhancements, future work is needed to test this important question directly. Indeed, our main purpose in developing the OC task was to enable such between-group studies since any conclusions about group differences need to be based on equating the degree of exposure to the enrichment objects. Such control is difficult with traditional environmental enrichment approaches.

In addition to new accounts for enrichment effects, we also propose a paradigm that is methodologically advantageous to existing enrichment paradigms. With current enrichment methods, it is not possible to have experimental control over the level of animal engagement with enrichment stimuli. This poses an issue when delivering enrichment with other experimental manipulations (genetic line, drug intervention, and sex), which may modulate animal engagement levels with enrichment stimuli. Conducting such manipulations, however, is crucial for maximizing the translational value of EE studies. With the OC, enrichment level can be matched across animals by fixing the duration spent on the course (as done in the current study) or the number of laps completed. Additionally, contrary to prior attempts of standardization^32,33^, the set-up of the course requires that the rodent interacts with each obstacle before proceeding through the track. Lastly, the OC has a control counterpart which approximates the degree of physical demand since the CC is identical in size, contains elevated hurdles recruiting muscular activity and energy expenditure, and both the control and experimental groups were run for a fixed duration. This is contrary to running wheels that do not systematically match the amount of exercise to that achieved in the enrichment cages.

For future OC-based studies, automation of the course can be simply achieved by inserting a trial-end gate that closes behind the animal once it reaches the reward site and a trial-start gate that opens on the other side of the reward site, and by automating reward delivery; both modifications can operate via a photo-sensitive beam for motion sensing. The trial-start gate could be used, for example, to control the number and timing of laps where control of lap number rather than total time is desired. Such automation would mitigate the labor-intensive nature of the OC and CC and add an additional degree of control.

Altogether, the present study provides a stepping-stone to better investigations of the therapeutic potential of enriched experience. Advancing knowledge of the effects of enrichment can provide a basis for lifestyle changes and improved therapies for disease states. Moreover, separating the biological effects of different components of EE is of importance because certain patient populations experiencing cognitive decline may also have physical inabilities (e.g., paralysis, motor impairment) and cannot benefit from the protective effects of physical activity-based EE but could achieve alternative forms of cognitive stimulation. Therefore, there is clinical value to understanding the cognitive enhancements of EE and more generally, expanding upon the current methods of studying enrichment effects in rodent models.

## Methods

### Overview

The study was composed of four groups of mice (*n* = 10 per group): obstacle course (OC), control course (CC), enrichment housing (EH), and standard housing (SH) groups. The course groups, both OC and CC, were housed the same as the SH group but were removed to run on their corresponding courses for 1 h/day, 6 days/week, for 9 weeks. Meanwhile, the EH mice remained in their enrichment cages, which were matched for object numbers with those of the OC. After the 9-week enrichment/control period, all animals were housed in cages intermediate in size to the SH and EH conditions without added enrichment while cognitive testing continued.

### Animals

A total of 40 28-day-old male C57BL/6 mice were obtained from Charles River (St. Constant, Quebec, Canada). All animals were 42 days old at the initiation of the study. After implementation of the 9-week enrichment protocols with their corresponding controls, animals were tested on a battery of object recognition and learning tasks beginning at 4 months of age. Tests were executed monthly for 4–10 months (Table 1).

Throughout the length of experimental testing, animals in the OC, CC, and SH groups were housed in clear polyethylene cages (16 cm × 12 cm × 26 cm) in groups of 3–4. Animals in the EH group were housed in larger cages (60 cm × 60 cm × 25 cm) in groups of 5. All cages were furnished with corncob bedding, crink-l’Nest, and cotton nest squares for maintenance of the quality of health of the mice. Animals were fed a lightly restricted diet of 3 g of food per day (Teklad Global 18% Protein Rodent Maintenance Diet, Harlan Teklad, WI) until their weight stabilized (6 weeks on the tracks).

Subsequently, all groups were transitioned to a limited diet of 2.5 g of the same food per day per mouse. Water was available for all groups ad libitum. Due to aggression, eight out of ten EH mice had to be individually housed immediately after the enrichment period, while only one out of ten animals from the OC group was individually housed 5 months post-discontinuation of enrichment. Animals were maintained on a 12-h light-dark cycle and tested during the light phase. All procedures followed the guidelines of the Canadian Council on Animal Care and were approved by the University of Guelph Animal Care Committee.

### Course set-up

The base tracks were custom-made at the University of Lethbridge and were identical for both the OC and CC groups (Fig. 1a, c). The track is a square-shaped design with the following dimensions: 860 mm arm length (including the corners) and 610 mm (excluding the corners), 120 mm arm width with surrounding walls of 70 mm in height on each side. The walls were utilized to contain the animal within the course. The track was mounted on four legs 18-cm in length. The track exterior was entirely enclosed by corrugated plastic cardboard, blocking the animal’s view of the testing room and limiting it to the interior of the course only. Corrugated barriers were also inserted at each corner to prevent the animal from cutting corners, ensuring passage through the course interior. Beam sensors were inserted in each corner and connected to a programmed Arduino board to track the time spent in each arm and the total time to complete each lap. Lastly, video monitoring was implemented using a top-down view of the mice for video recording. Offline video analysis using DeepLabCut was implemented for animal tracking (Supplementary Videos 3–4). Gray rectangular board pieces made of sintra foam sheets were used as a base for obstacles, and a total of 12 inserts (base+obstacle) filled the course (three inserts per arm) at a given time. A 0.03 ml drop of Neilson ® strawberry milkshake (fat/lipid content: 22.58 g/L, sugar content: 125.81 g/L) was given as a reward at the end of each lap for both OC and CC groups. A wall divider was placed immediately before the start site of the course to ensure unidirectional animal movement.

### Obstacle course protocol

Each arm of the track was allocated three obstacles positioned serially. Handmade obstacles were glued to the gray plastic bases to fit within the course perimeter. Daily, within each group (OC vs. CC), mice were run in a randomized order (between first to last) for a 1-h duration over the course of 9 weeks. A detailed protocol was followed for each week, which ensured matched course alterations across animals. The protocol can be found in the supplementary material (Supplementary Fig. 1) and is briefly outlined here.

All groups were habituated to handling and the OC and CC groups to food reward (strawberry milkshake) for 1 week. In the following week, animals were gradually habituated to an empty course for the first 4 days. In the subsequent 2 days, 12 triangular hurdles were gradually inserted to fill the entire course (Fig. 1c and Supplementary Fig. 1). Course enrichment began after an empty course and hurdle habituation. In the first week of enrichment, three obstacles were added each day for 4 days until the course contained 12 obstacles. In the subsequent days for 8 weeks, the following daily manipulations were implemented: change to existing obstacle→ add new obstacle → change to existing obstacle → add new obstacle → change to an existing obstacle. This led to a daily protocol of adding two new obstacles and making three rearrangements to the track (Fig. 1e and Supplementary Fig. 1). Changes consisted of rotating obstacles 180 degrees in their position or swapping the positions of a given pair of obstacles. These manipulations were spread across the 1-h session at intervals of 10 min. Supplementary Video 2 illustrates a segment of an OC session with obstacle manipulations. OC mice experienced a total of 132 obstacles in the 9-week enrichment period (Supplementary Video 1) and 12 identical hurdles in the week prior to enrichment.

### Control course protocol

The CC group was habituated in the same manner as described above. Like the OC, the CC was gradually filled with 12 hurdles. There were no subsequent manipulations performed on the course. The CC group ran the 12-hurdle-filled course for a matched duration of 1-h for the remainder of the study. This group served as a control for physical exercise.

### Home-cage enrichment protocol

Larger-sized cages (60 cm × 60 cm × 25 cm) were used for the EH group. Home-cage enrichment began on the same day as course enrichment. The addition of new objects followed the same daily pattern as the addition of new obstacles for the OC group. Three objects were added to cages daily for 3 days, followed by the replacement of two old objects with two new ones. The EH group experienced a total of 132 objects throughout the duration of the enrichment period. Kraft Smooth peanut butter was put in different locations in the enriched cages to facilitate for aging and interaction with enrichment objects. In contrast, the SH cage enrichment was limited to nesting material with no additional objects or cage manipulations.

### Behavioral testing

Mice were tested on six behavioral protocols, five of which exploit rodents’ tendency to explore novel items preferentially^53,55^ and the sixth involved a touchscreen-based learning and reversal task^56^. All tests except the object location and touchscreen tasks were conducted using an enclosed Y-shaped apparatus^39^. Each object was used once for each object-based task. Table 1 indicates the timeline for each behavioral test. Objects were always washed with 50% ethanol solution immediately prior to being placed in the apparatus. These tasks were chosen to assess different complementary aspects of an object and spatial cognitive processing and learning, partly as an index of the generalizability of the beneficial effects of the enrichment protocols. The principal behavioral score for object-based tests was an object discrimination ratio (DR), the difference in time spent exploring the novel versus the familiar object divided by the sum (DR = time of novel object exploration – time of familiar object exploration/total time of exploration). DR values were calculated for each mouse and then averaged across mice. Exploration times during sample and choice phases for all object tasks as well as significant differences in exploration times between groups are summarized in Supplementary Table 1 and its corresponding text. Pearson’s correlations between sample/choice exploration times and choice DRs for all object tasks are summarized in Supplementary Table 2, with significant results reported in the corresponding text. For the pairwise discrimination task, trial touch and reward acquisition latency analyses are summarized in Supplementary Fig. 3.

### Spontaneous object recognition (SOR)

The SOR task^47^ was used to assess whether EE-mediated memory enhancements for object identity can be induced with the OC experience. SOR testing was initially implemented during the 9th week of enrichment and again 1-month following discontinuation of enrichment. The task consisted of a sample (7 or 10 min) phase, during which animals were presented with two identical objects in each arm of the Y-apparatus (Fig. 2a). This was followed by a delay, and then the choice phase (3 min), in which one of the two objects from the sample phase was replaced with a novel object. Two delay durations were tested for each mouse, 20-min and 24-h, in order to assess short and long-term memory, respectively. The novel object position was counterbalanced between the right and left arms of the Y-apparatus.

### Object location memory (OLM)

The OLM task was used to test whether OC enrichment was sufficient to induce enhancements of spatial memory, independently of object identity^47^ OLM testing was implemented 5 and 6 months post enrichment and consisted of a sample (10 or 7 min), delay (20 min or 24 h), and choice phases (3 min) (Fig. 3a). The task is run in an open field (45 × 45 × 30 cm). In the sample phase, two identical objects are presented in two corners of the open field. Following the retention delay, the same identical objects are presented, but one is displaced to the opposite corner. Preference for this displaced object indicates recognition of the original spatial location.

### Two-choice pairwise visual discrimination (PD) with reversal

The PD task was used to replicate EE enhancements in cognitive flexibility^38^ and to assess learning capacity^56^ using a non-object-based task. This is a ten-stage protocol, with the first seven stages aimed at gradually habituating the mouse to the experimental set-up (Supplementary Fig. 2), and stages 8–10 involved learning image-reward associations as well as the reversal of such associations (Fig. 4a). Each mouse was required to reach a pre-defined criterion to proceed to the subsequent stage in the experiment. Stage 1 is a 10 min habituation to the chamber. Stage 2 is a 20 min habituation, including habituation to simultaneous light, tone, and strawberry milkshake reward delivery. Stage 3 is the same as 2, with an extension to 40 min. Stage 4 involved delivering reward/tone/light upon the mouse touching the singly presented stimulus. Stage 5 required the mouse to touch the presented image for reward/tone/light delivery. If the criterion is not reached after a maximum of seven sessions, the animal is taken back to stage 4. Stage 6 required the mouse to initiate a subsequent trial via a nose poke of the food tray. For stages 4–6, the criterion was set as completion of 30 trials in a 60-min duration, with a total of 40 images utilized. Stage 7 introduced punishment for touching the incorrect location, where a 5-s time-out was introduced with the chamber light turned on before the 20-sec inter-trial interval (ITI), and a subsequent trial could be initiated. Criterion was set as completion of 24 out of 30 trials or better within a 60-min duration for two consecutive sessions, and two stimuli were utilized. Stages 8–9 involved presentation of both stimuli, delivery of reward upon animal contacting an S+ stimulus, and punishment following the S− stimulus contact (Fig. 4a.i). A correct response is followed by a new trial, and incorrect response is followed by a correction trial where the same trial display is repeated until the correct response is achieved. Criterion was set to a correct response for at least 24 out of 30 trials or better within 60 min for two consecutive sessions. Correction trials were not counted toward criterion. Stage 10 involved contingency reversal where responding to the previously learned S− becomes associated with reward (S+) and vice versa (Fig. 4a.ii). The session ended after the completion of 30 trials or 60-min duration.

### Object category recognition (OCR)

The OCR task was used to evaluate the ability to generalize within object categories and distinguish between separate categories^57^. OCR testing was implemented between 1 and 2 months post-discontinuation of enrichment and consisted of a sample (10 min), delay (30 min, 1 h), and choice phases (3 min) (Fig. 5a). In the sample phase, mice were exposed to two different objects of the same category (e.g., toy cars). In the choice phase, two new objects were introduced: a new object from the same category and a new object from a different category. Preferential exploration of the new object from the previously unsampled category was interpreted as a within-category generalization (i.e., a preference for the novel category).

### View-invariant object recognition (VIOR)

The VIOR task was used to assess the flexible use of object representations when mice were presented with previously explored objects from novel perspectives^47^. VIOR testing was implemented 3 months post-discontinuation of enrichment and consisted of a sample (10 min), delay (5 min), and choice phases (3 min) (Fig. 5c). In the sample phase, two identical objects were shown in the same orientation. For the choice phase, one object was rotated (180 degrees) while the other remained in its original orientation. In the case of the VIOR task, the absence of preference for either object in the choice phase is interpreted as a recognition that the two objects are the same despite their differing orientations.

### Cross-modal object recognition (CMOR)

The CMOR task was utilized to test object recognition across sensory modalities^47^. CMOR testing was implemented 4 months post-discontinuation of enrichment and consisted of a sample (10 min), delay (30-min, 1 h), and choice phases (2 min) (Fig. 6a). In the tactile sample phase, mice were exposed to two identical objects, one in each arm, under red light (to prevent visual processing). In the visual choice phase, transparent barriers were inserted in each arm between the mouse and the object, preventing tactile interaction. Preference for the novel object in the choice phase thus indicates cross-modal (tactile-to-visual) object recognition.

### General behavioral procedure and statistical analysis

Regarding the OR tasks, the behavioral testing room, Y-maze apparatus, video-scoring procedure, and statistical analyses were identical to those described in Creighton et al., 2019. To clarify further, for all object tasks, a DR was calculated for both the sample and choice phases. Using SPSS IBM, performance on recognition tasks was assessed using either one way or mixed analysis of variance (ANOVA) where appropriate. Independent t-tests were used to further assess the differences between groups. Bonferroni corrections were applied for multiple comparisons. Using paired-samples *t*-tests, the choice phase DR was then compared to the sample phase DR to assess whether mice discriminated significantly above chance. Additionally, for these tasks, each animal underwent an individual trial. However, for the OLM, OCR, and VIOR tasks, high variance was observed, and each animal experienced a second trial, where a single mean value across the two trials was obtained per animal.

The visual discrimination and reversal tasks were conducted in automated touchscreen operant chambers (Bussey-Saksida Mouse Touchscreen Chamber System, Lafayette Instrument Co., Lafayette, IN), with instructions and event recordings operated through the software Whisker Server and ABET II (Campden Instruments Ltd, Loughborough, England).

For PD task statistical analysis, a repeated measures four-way analysis of variance (ANOVA) was used to analyze between-group differences across the relevant testing days. Based on significant differences identified, appropriate post hoc analysis was performed sequentially. All other group comparisons were completed with a one-way ANOVA and appropriate post hoc tests.

### Reporting summary

Further information on research design is available in the Nature Research Reporting Summary linked to this article.

## Supplementary information

Supplementary Material

Supplementary Video 1 | Example obstacles.

Supplementary Video 2 | Example obstacle course session.

Supplementary Video 3 | Deep learning tracking of animal performance on the OC

Supplementary Video 4 | Deep learning tracking of animal performance on the CC

Reporting Summary

## Data Availability

The data that support the findings of this study are available from the corresponding authors upon reasonable request.

## Code availability

Custom codes generated for this study are available on request from S.G.
