# Supplementary material for: A rodent obstacle course procedure controls delivery of enrichment and enhances complex cognitive functions
Source: NPJ Sci Learn. 2022 Sep 3;7:21. doi: 10.1038/s41539-022-00134-x (PMC9440923; doi:10.1038/s41539-022-00134-x)
Supplement: Supplementary file 1 — Supplementary Material [file 41539_2022_134_MOESM1_ESM.docx]

**Supplementary Material**


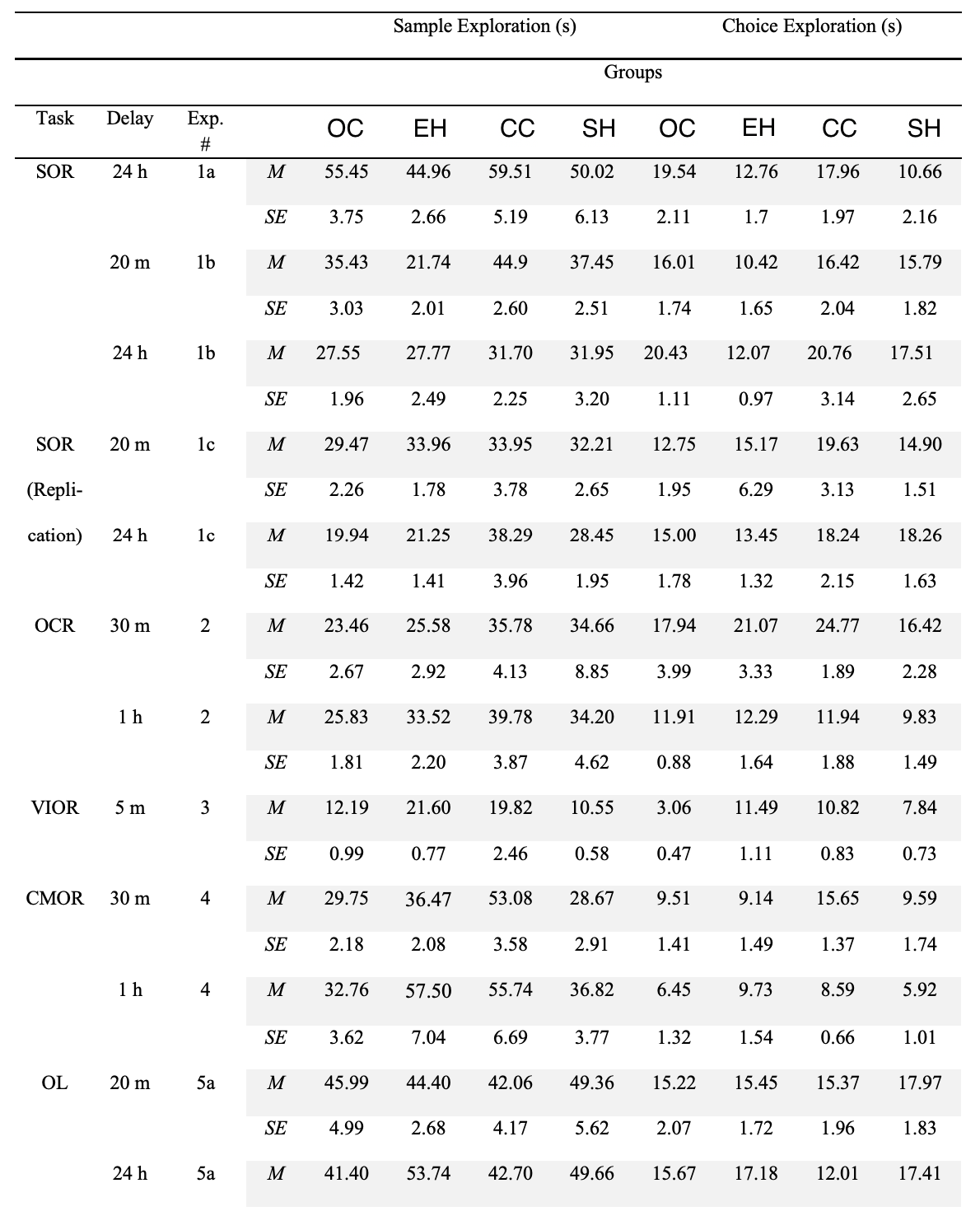


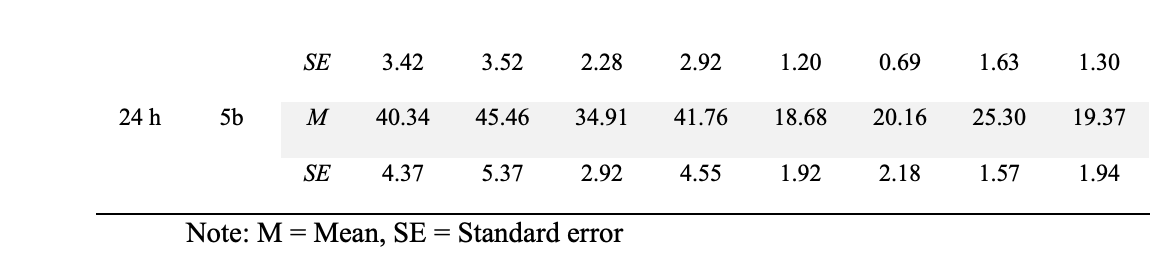


**Supplementary Table 1. Object recognition exploratory behavior for all object tasks**

Associated significant results regarding exploration time

- Experiment 1a:
  - A one-way ANOVA found a significant effect of group for choice phase exploration, *F*(3, 36) = 4.432, *p* = 0.009, partial h2 = 0.270.
- Experiment 1b:
  - For sample phase exploratory behaviour, a repeated measures ANOVA found a significant interaction between delay and group, *F*(3, 36) = 4.687, *p* = 0.007 , partial h2 = 0.281, as well as a main effect of delay, *F*(1, 36) = 7.551, *p* = 0.009 , partial h2 = 0.173, and group, *F*(3, 36) = 11.228, *p* = 0.009 , partial h2 = 0.438.
  - For choice exploration, a main effect of delay, *F*(1, 36) = 4.337, *p* = 0.044 , partial h2 = 0.108, and group, *F*(3, 36) = 6.015, *p* = 0.002, partial h2 = 0.334, was found.
- Experiment 1c:
  - For sample exploration, a repeated measures ANOVA revealed a significant interaction between delay and group, *F*(3, 36) = 7.648, *p* < 0.001 , partial h2 = 0.389, as well as a significant main effect of delay, *F*(1, 36) = 16.032, *p* < 0.001, partial h2 = 0.308, and group, *F*(3, 36) = 4.934, *p* = 0.006 , partial h2 = 0.291.
- Experiment 2:
  - A repeated measures ANOVA revealed a significant effect of group, *F*(3, 36) = 3.029, *p* = 0.042, partial h2 = 0.202, on sample exploration.
  - A significant main effect of delay, *F*(1, 36) = 29.059, *p* < 0.001, partial h2 = 0.447, was found on exploration during the choice phase.
- Experiment 3:
  - A one-way ANOVA revealed a significant effect of group, *F*(3, 36) = 15.170, *p* < 0.001, partial h2 = 0.558 on sample phase exploration, and choice phase exploration, *F*(3, 36) = 22.055, *p* < 0.001, partial h2 = 0.648.
- Experiment 4:
  - A significant main effect of delay, *F*(1, 36) = 7.163, *p* = 0.011, partial h2 = 0.116, and group, *F*(3, 36) =12.116, *p* < 0.001, partial h2 = 0.502 was found on sample exploration using a repeated measures ANOVA.
  - A significant interaction between delay and group, *F*(3, 36) =3.484, *p* = 0.026, partial h2 = 0.225, and main effect of delay, *F*(1, 36) = 15.395, *p* < 0.001, partial h2 = 0.300, and group, *F*(3, 36) =3.579, *p* = 0.023, partial h2 = 0.230 were found on exploration during the choice phase.


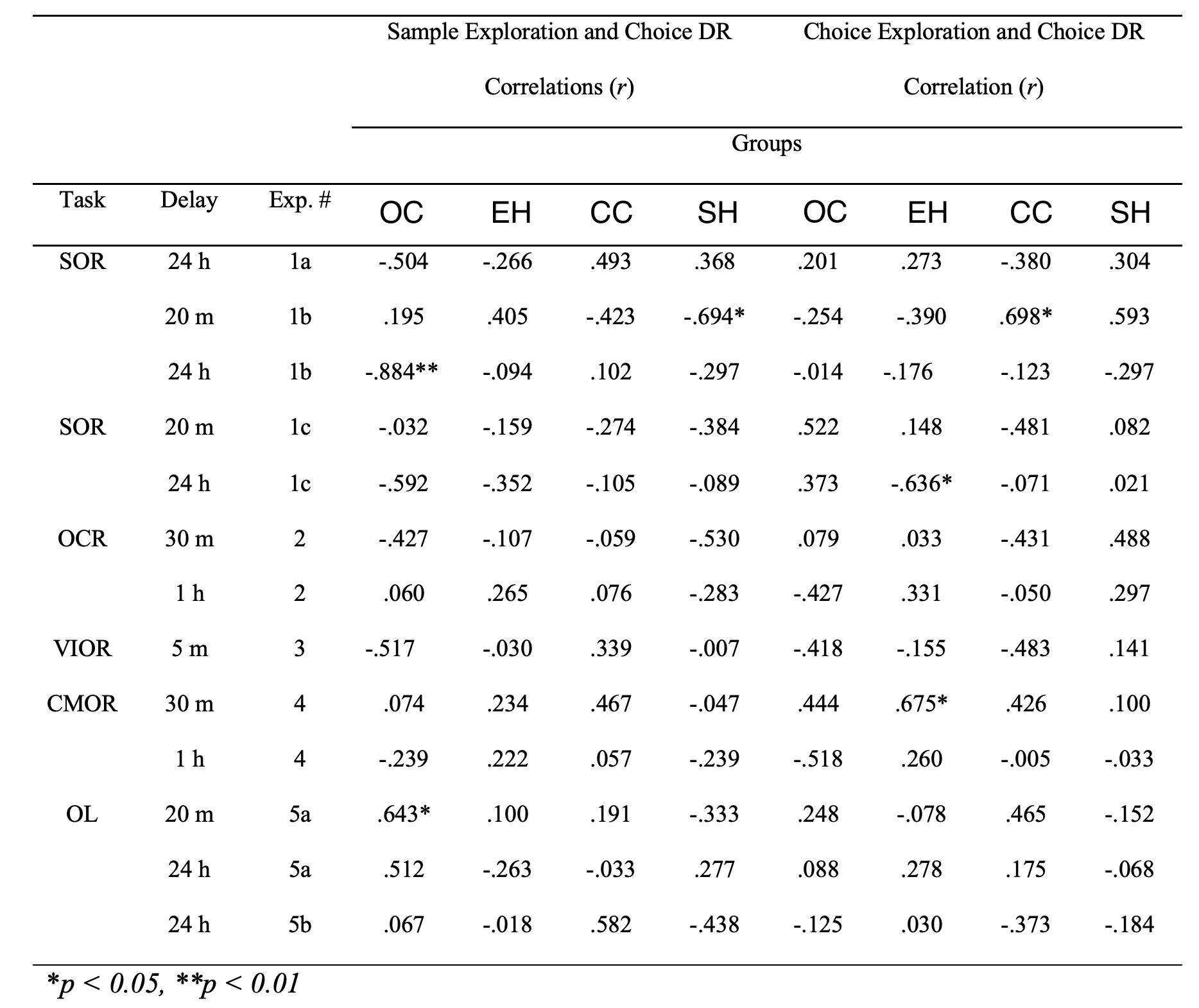


**Supplementary Table 2. Pearson correlations between sample and choice exportation and choice discrimination ratio (DR) for all object tasks**

Associated significant results regarding exploration time

Positive and negative relationships with exploration time and discrimination

1. Experiment 1b: Significant negative correlation between sample exploration and choice DR for the sub-optimal SOR task with the 20-min delay for mice in the SH group, *r*(10) = -0.694, *p* = 0.026.
   1. More exploration associated with lower DR for SH
2. Experiment 1b: Significant negative correlation between sample exploration and choice DR for the sub-optimal SOR task with the 24-hr delay for mice in the OC group, *r*(10) = -0.881s, *p* = 0.001
   1. More exploration associated with lower DR for OC
3. Experiment 1c: Significant negative correlation for the sub-optimal SOR task with the 24-hr delay for mice in the EH group when this task was performed 1 month post-enrichment, *r*(10) = -0.636, *p* = 0.048.
   1. More exploration associated with lower DR for EH
4. Experiment 1b: Significant positive correlation between choice exploration and choice DR for the sub-optimal SOR task with the 20-min delay for mice in the CC group, *r*(10) = 0.698, *p* = 0.025
   1. More exploration associated with higher DR for CC
5. Experiment 4: Significant positive correlation between choice exploration and choice DR was found for the CMOR task with the 30-min delay for EH group, *r*(10) = 0.675, *p* = 0.032.
   1. More exploration associated with higher DR for EH
6. Experiment 5a: Significant positive correlation between total sample exploration and choice DR with the OL task with the 20-min delay for mice in the OC group, *r*(10) = 0.643, *p* = 0.045.
   1. More exploration associated with higher DR for OC


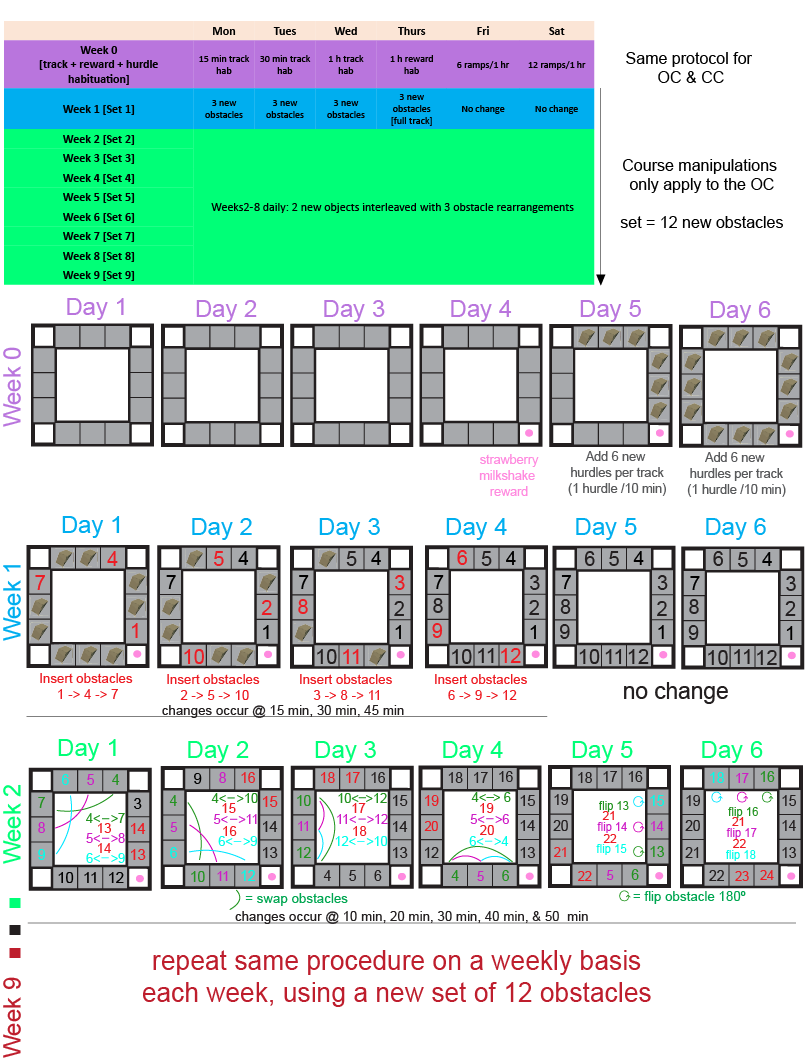


**Supplementary Figure 1 | Detailed OC enrichment protocol.**

Top panel is a table outlining OC and CC protocols including weekly and daily executions. Bottom schematics further detail the daily and weekly manipulations to the track. Habituation occurred at week 0 in the same manner for both the OC and CC groups. Weeks 1 – 9 apply to the OC group only. By the end of week 1, the OC was filled with novel obstacles. The manipulations that took place on week 2 were done in a similar manner for weeks 3 - 9. By the end of each week, a full new set of 12 obstacles were introduced to the OC track. Enrichment occurred for a duration of 9 weeks. Each day, mice within each group were run on their respective tracks in a randomized order between one and 10 (first to last).


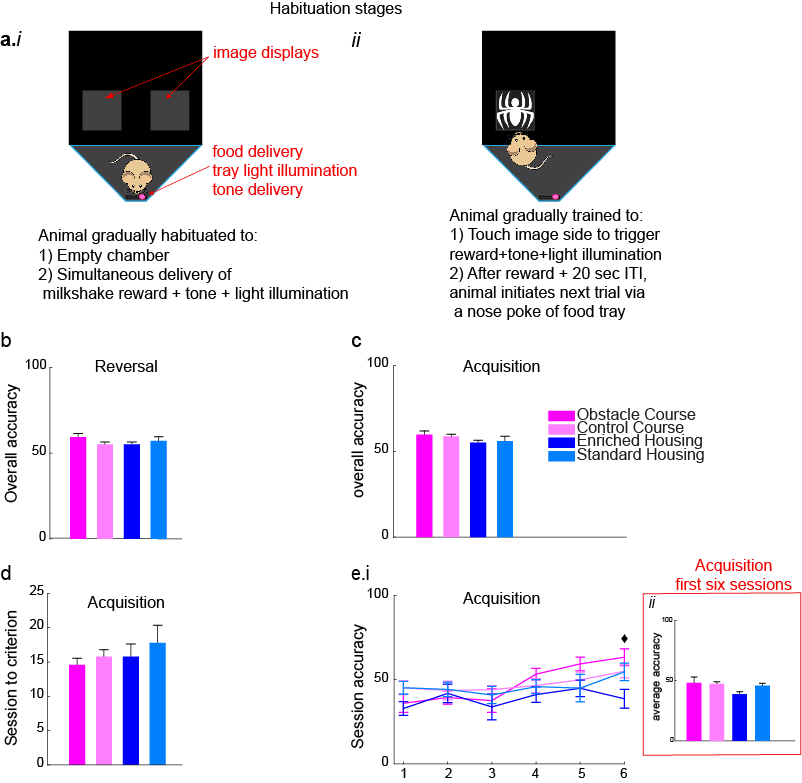


**Supplementary Figure 2 | PD task – habituation schematic and supplementary results**

**a.** Schematic representation of the PD habituation stages. **a.i-ii.** Gradually habituating the animal to: **i**. recording chamber, simultaneous delivery of reward, tone, and light, **ii.** touching a presented stimulus to trigger reward, and nose-poke in reward tray to trigger stimulus presentation. **b-c.** Mean accuracy across all **(b)** reversal F(3, 36) =1.196, *p* = 0.325**,** partial η^2^ = 0.091 and **(c)** acquisition F(3, 36) = 1.945, *p* = 0.14, partial η^2^ = 0.139 sessions per group. **d.** Mean session number to reach criterion (performance of 80% accuracy on two successive sessions) per group in the acquisition phase. No group main effect was observed F(3,36) = 2.004, *p* = 0.131, partial η^2^ = 0.143. **e.i.** Group percent accuracy on the first six acquisition sessions with a main effect of session, F(5, 36) = 6.975, *p* < 0.001, partial η^2^ = 0.162 and an effect of group in session six, F(3,36) = 4.340, p = 0.010, partial η^2^ = 0.266, with the OC group performing significantly higher than EH on session six *t*(18) = 3.294, *p* = 0.004. ***ii.*** Mean accuracy across the first six acquisition sessions for each group revealed no significant differences in group accuracy, F(3,36) = 1.78, *p* = 0.168, partial η^2^ = 0.129. SEM represent standard error of the mean across animals.

**Supplementary Figure 3 | PD task – trial touch and reward collection latencies**

**a-b.** Mean **(a)** acquisition, F(3, 28) =1.353, *p* = 0.277**,** partial η^2^ = 0.127 and **(b)** reversal F(3, 19) =1.154, *p* = 0.353**,** partial η^2^ = 0.154 trial touch latencies. A single latency value was obtained per animal reflecting the mean latency across correct and incorrect trials from the first 6 and 5 sessions for acquisition and reversal, respectively. **c-d.** Same as in **(a-b)**, but for reward collection latency (correct trials) for **(c)** acquisition, F(3, 29) = 0.855, *p* = 0.475**,** partial η^2^ = 0.081 and **(d)** reversal, F(3, 19) =1.016, *p* = 0.408**,** partial η^2^ = 0.138. SEM represent standard error of the mean across animals. No group main effects were observed for acquisition and reversal trial touch and reward collection latencies.

**Supplementary Video 1 | Example obstacles.**A video displaying utilized obstacles for the OC.

**Supplementary Video 2 | Example obstacle course session.**

Example Obstacle Course session from week 5, day 1, animal # 13. Demonstrated in the video are 3 consecutive runs on the OC by the same mouse: one lap prior to and two laps following course manipulation. As demonstrated, animals tended to take longer time to complete laps immediately following course manipulation, spending more time interacting with the manipulated or new obstacle. However, animal speed returned to baseline on the second lap after manipulation. In this particular video, the manipulation involved swapping two already existing obstacles.

**Supplementary Video 3-4 | Deep learning tracking of animal performance on the OC (Video 3) and CC (Video 4).**

Using image frames extracted from video monitoring of animals on the course, DeepLabCut was trained to track animal position (cyan circle) in order to calculate time to complete laps on the respective tracks. Note: consent was obtained from the researcher viewable in Supplementary Video 4 for publication of the video.
