# Supplementary material for: A rodent obstacle course procedure controls delivery of enrichment and enhances complex cognitive functions
Source: NPJ Sci Learn. 2022 Sep 3;7:21. doi: 10.1038/s41539-022-00134-x (PMC9440923; doi:10.1038/s41539-022-00134-x)
Supplement: Supplementary file 6 — Reporting Summary [file 41539_2022_134_MOESM6_ESM.pdf]

nature portfolio

Corresponding author(s): Sandra Gattas
Bruce L. McNaughton
Boyer D. Winters

Last updated by author(s): 01/11/22

Reporting Summary

Nature Portfolio wishes to improve the reproducibility of the work that we publish. This form provides structure for consistency and transparency in reporting. For further information on Nature Portfolio policies, see our Editorial Policies and the Editorial Policy Checklist.

Statistics

For all statistical analyses, confirm that the following items are present in the figure legend, table legend, main text, or Methods section.

n/a	Confirmed	
☒	☐	The exact sample size (*n*) for each experimental group/condition, given as a discrete number and unit of measurement
☒	☐	A statement on whether measurements were taken from distinct samples or whether the same sample was measured repeatedly
☒	☐	The statistical test(s) used AND whether they are one- or two-sided<br>*Only common tests should be described solely by name; describe more complex techniques in the Methods section.*
☒	☐	A description of all covariates tested
☒	☐	A description of any assumptions or corrections, such as tests of normality and adjustment for multiple comparisons
☒	☐	A full description of the statistical parameters including central tendency (e.g. means) or other basic estimates (e.g. regression coefficient) AND variation (e.g. standard deviation) or associated estimates of uncertainty (e.g. confidence intervals)
☒	☐	For null hypothesis testing, the test statistic (e.g. *F*, *t*, *r*) with confidence intervals, effect sizes, degrees of freedom and *P* value noted<br>*Give P values as exact values whenever suitable.*
☒	☐	For Bayesian analysis, information on the choice of priors and Markov chain Monte Carlo settings
☒	☐	For hierarchical and complex designs, identification of the appropriate level for tests and full reporting of outcomes
☒	☐	Estimates of effect sizes (e.g. Cohen's *d*, Pearson's *r*), indicating how they were calculated

Our web collection on statistics for biologists contains articles on many of the points above.

Software and code

Policy information about availability of computer code

Data collection	Custom made arduino code was used to program sensors in the obstacle course track to track lap times and to export data as csv files. The visual discrimination and reversal task instructions and event recordings were operated through the software Animal Behaviour Environment Test (ABET) II (Campden Instruments Ltd, Loughborough, England).
Data analysis	Custom made MATLAB code was used to analyze the track sensor data. DeepLabCut was implemented for animal tracking in the offline video analysis. All analyses of object task data was conducted using SPSS 26.0 or later.

For manuscripts utilizing custom algorithms or software that are central to the research but not yet described in published literature, software must be made available to editors and reviewers. We strongly encourage code deposition in a community repository (e.g. GitHub). See the Nature Portfolio guidelines for submitting code & software for further information.

Data

Policy information about availability of data

All manuscripts must include a data availability statement. This statement should provide the following information, where applicable:

- Accession codes, unique identifiers, or web links for publicly available datasets
- A description of any restrictions on data availability
- For clinical datasets or third party data, please ensure that the statement adheres to our policy

Data is available upon request from corresponding authors.

Field-specific reporting

Please select the one below that is the best fit for your research. If you are not sure, read the appropriate sections before making your selection.

☐ Life sciences ☒ Behavioural & social sciences ☐ Ecological, evolutionary & environmental sciences

For a reference copy of the document with all sections, see nature.com/documents/nr-reporting-summary-flat.pdf

Behavioural & social sciences study design

All studies must disclose on these points even when the disclosure is negative.

Study description	quantitative-experimental
Research sample	40 28-day-old male C57BL/6 mice<br>The sample is representative of male C57BL/6 mice<br>This was an ideal sample as the goal of the study was to advance the rodent enrichment mode. Given the labor-intensive nature of the experiments and the abundance of male-only studies in the EE literature we opted for a male only sample in this initial study.
Sampling strategy	random<br>Power calculations indicate a sample size of 12, however we opted for a sample size of 10 given the labor intensive nature of the experiments.
Data collection	Built-in track sensors and video monitoring were used collect track behavioral data.<br>Video monitoring was used to collect object task data. These videos were analyzed with custom software which was used to score exploration duration (s) of objects during exploratory bouts. The touchscreen task instructions and event recordings were operated through the software Whisker Server and ABET II.<br>Exported track data was subsequently analyzed using custom code in MATLAB and using DeepLabCut. Object videos were then analyzed offline by researchers. Only researchers were present during the experiments. Researchers were not blinded to the experimental group.
Timing	this is detailed in table 1
Data exclusions	no data were excluded from the analyses
Non-participation	N/A
Randomization	*If participants were not allocated into experimental groups, state so OR describe how participants were allocated to groups, and if allocation was not random, describe how covariates were controlled.*

Reporting for specific materials, systems and methods

We require information from authors about some types of materials, experimental systems and methods used in many studies. Here, indicate whether each material, system or method listed is relevant to your study. If you are not sure if a list item applies to your research, read the appropriate section before selecting a response.

Materials & experimental systems

n/a	Involved in the study	
☒	☐	Antibodies
☒	☐	Eukaryotic cell lines
☒	☐	Palaeontology and archaeology
☐	☒	Animals and other organisms
☒	☐	Human research participants
☒	☐	Clinical data
☒	☐	Dual use research of concern

Methods

n/a	Involved in the study	
☒	☐	ChIP-seq
☒	☐	Flow cytometry
☒	☐	MRI-based neuroimaging

Animals and other organisms

Policy information about studies involving animals; ARRIVE guidelines recommended for reporting animal research

Laboratory animals	28-day-old male C57BL/6 mice
Wild animals	The study did not involve wild animals

Field-collected samples	The study did not involve samples collected from the field
Ethics oversight	All procedures followed the guidelines of the Canadian Council on Animal Care and were approved by the University of Guelph Animal Care Committee

Note that full information on the approval of the study protocol must also be provided in the manuscript.
